# A frequent CYP2D6 variant promotes skipping of exon 3 and reduces CYP2D6 protein expression in human liver samples

**DOI:** 10.3389/fphar.2023.1186540

**Published:** 2023-07-27

**Authors:** Joseph M. Collins, Hannah Lester, Samia Shabnaz, Danxin Wang

**Affiliations:** Department of Pharmacotherapy and Translational Research, Center for Pharmacogenomics, College of Pharmacy, University of Florida, Gainesville, FL, United States

**Keywords:** cytochrome P450s, CYP2D6, single nucleotide polymorphisms, drug metabolism, genetic test, metabolic phenotype, splice variant, protein expression

## Abstract

CYP2D6 is one of the most polymorphic drug-metabolizing enzymes in the liver. While genetic *CYP2D6* variants serve as clinical biomarkers to predict CYP2D6 activity, large inter-person variability in CYP2D6 expression remains unaccounted for. Previous results suggest that there is variable expression of a CYP2D6 splice isoform with an in-frame deletion of exon 3 (CYP2D6ΔE3) encoding a protein lacking numerous active site residues. Here, using fragment analysis and RT-qPCR, we revealed that rs1058164 G (MAF = 27%–43%) is associated with increased formation of CYP2D6∆E3 in human liver samples (1.4–2.5-fold) and transfected cells. Furthermore, western blots showed that rs1058164 G was associated with a 50% decrease in full-length hepatic CYP2D6 protein expression. In addition, by studying a larger liver cohort, we confirmed our previous results that rs16947 (CYP2D6*2) reduces full-length CYP2D6 mRNA by increasing the production of an unstable splice isoform lacking exon 6 (CYP2D6ΔE6) and that the impact of CYP2D6ΔE6 is offset in carriers of the downstream enhancer variant rs5758550. The three frequent SNPs (rs1058164, rs16947, and rs5758550) form various 3-SNP-haplotypes, each with distinct CYP2D6 expression characteristics. Using an expression score (ES) system, we tested the impact of the 3-SNP-haplotype on improving the standard model to predict hepatic CYP2D6 protein expression based on genotype. A model that incorporates the 3-SNP-haplotype provided the best fit for CYP2D6 expression and also accounted for more variability in CYP2D6 protein levels (59%) than a model based on the accepted standard (36%) or one that only adds rs16947 and rs5758550 (42%). Clinical studies are needed to determine whether including the 3-SNP-haplotype alongside current standard CYP2D6 models improves the predictive value of CYP2D6 panels.

## 1 Introduction

As one of the most important drug-metabolizing cytochrome P450 (CYP) enzymes in the liver CYP2D6 metabolizes ∼25% of currently used medications. CYP2D6 is highly polymorphic, and there are over 100 CYP2D6 star (*) alleles (PharmVar, https://www.pharmvar.org). In addition to CYP2D6 inhibitors, genetic variants are a primary cause of the large inter-person variability in CYP2D6 enzymatic activity (Ingelman-Sundberg, 2005) and profoundly impact the therapeutic outcomes of CYP2D6 substrate drugs (Goetz et al., 2018; Brown et al., 2019). CYP2D6 genetic testing has become a useful tool for personalized drug therapy and the Clinical Pharmacogenomics Implementation Consortium (CPIC) has published guidelines for using CYP2D6 genetic information to guide therapy for many medications (Crews et al., 2014; Hicks et al., 2015; Bell et al., 2017; Hicks et al., 2017; Goetz et al., 2018).

Nine common star alleles (*1, *2, *3, *4, *9, *10, *17, *29, and *41) and gene copy number variations (CNVs) (e.g., gene deletion (*5), gene duplication and multiplications) are included in most CYP2D6 panels. An activity score (AS) system (Gaedigk et al., 2008) is commonly employed to predict the enzymatic activity of CYP2D6 based on star allele genotype according to the CPIC standards (Crews et al., 2014; Hicks et al., 2017), which is then translated into CYP2D6 metabolizer phenotypes, including poor metabolizers, intermediate metabolizers, normal metabolizers, and ultra-rapid metabolizers (Crews et al., 2014; Hicks et al., 2017). With the exception of poor metabolizers, the relationship between CYP2D6 genotype and phenotype remains ambiguous, with large variability within genotype/haplotype groups (Pedersen et al., 2005; Gaedigk et al., 2008; Llerena et al., 2012; Montané Jaime et al., 2013), suggesting that the full complexity of genetic CYP2D6 variation remains uncertain. Numerous rare genetic changes have been identified in CYP2D6 through the extensive genome, exome, and long-read sequencing (Nofziger et al., 2019), but their rarity precludes their ability to explain population-level variation in CYP2D6. In particular, observed variability in the expression of CYP2D6 at both the mRNA and protein levels has not been fully characterized. Since CYP2D6 is not inducible by environmental factors (Bock et al., 1994), it appears that the likely cause for differences in CYP2D6 expression arises from cis-acting genetic factors, as opposed to trans-acting factors.

We previously identified two interacting variants that regulate the expression of CY2D6: an enhancer variant (rs5758550) that increases CYP2D6 transcription and an exonic variant rs16947 (core SNP of *2) that promotes an alternate and unstable splice isoform missing exon 6, which is out-of-frame and undergoes nonsense-mediated decay, thereby reducing expression of full-length CYP2D6 (Wang et al., 2014; Wang et al., 2015). The combination of rs16947 and rs5758550 form alternate CYP2D6 haplotypes with varying enzymatic activities and occur at different frequencies in different populations (Wang et al., 2014; Ray et al., 2019; Elias et al., 2020). In a recent cross-ancestry GWAS, the compensatory effect of rs5758550 on the deleterious effects of rs16947 was confirmed for CYP2D6-dependent endoxifen plasma concentration (Khor et al., 2023). However, there are inconsistent results regarding the impact of rs5758550 and rs16947 on *in vivo* CYP2D6 protein expression/activity (Sanchez-Spitman et al., 2017; Ning et al., 2018; Thomas et al., 2020; Dinh et al., 2022; Khor et al., 2023). Furthermore, overall CYP2D6 mRNA levels do not seem to correlate well with protein levels (Ning et al., 2018), indicating that additional uncharacterized post-transcriptional events influence CYP2D6 expression. In this study, we identified a frequent synonymous variant rs1058164 that is associated with increased formation of a CYP2D6 splice isoform missing exon 3 (CYP2D6∆E3). Based on LD structure and different allele frequencies, rs5758550, rs16947, and rs1058164 form various 3-SNP haplotypes in different populations (Machiela and Chanock, 2015). Therefore, we evaluated their combined effects by considering the different 3-SNP haplotypes on full-length CYP2D6 expression in a liver sample cohort derived from both European American (EA) and African American (AA) donors (*n* = 244). Our results demonstrated that including all known regulatory SNPs better predicted full-length CYP2D6 protein expression in the liver samples.

## 2 Materials and methods

### 2.1 DNA and RNA preparation from liver tissue samples

Human liver samples (121 EA and 123 AA) were obtained from The Cooperative Human Tissue Network under an exempted protocol approved by the University of Florida IRB committee. Liver sample characteristics are published (Collins and Wang, 2021a). Biopsy liver tissues were immediately frozen in liquid nitrogen, shipped on dry ice, and stored at −80°C until use. Genomic DNA was prepared using DNeasy Blood & Tissue kits (Qiagen, United States). Total RNA was prepared from liver samples using direct-zol RNA miniprep plus kits (Zymos Research, CA, United States). RNA quality control and cDNA synthesis were performed as described (Collins and Wang, 2021b).

### 2.2 Genotyping

CYP2D6 SNPs were genotyped using PCR amplification of the entire CYP2D6 gene (∼5 kb) followed by multiplex primer extension assays (SnapShot assays), as described (Sistonen et al., 2005). Twelve SNPs (*10—rs1065852, *17—rs28371706, *6—rs5030655, *4—rs3892097, *3—rs35742686, *9—rs5030656, *2—rs16947, *39—rs1135840, *41—rs28371725, *29—rs59421388, rs5758550, and rs1058164) were genotyped (see Supplementary Table S1 for primers and probes). Copy number variation (CNV) was genotyped using TaqMan copy number variation assays with two probes targeting intron 6 and exon 9 of CYP2D6 (CNV assay information is in Supplementary Table S1). Consistent results from both probes were required for determining CNV, as samples with inconsistent results may carry hybrid genes, which were subsequently excluded from further analysis. The genotype (or haplotype) of duplicated genes was assigned by comparing the allelic ratio of CNV samples to the average allelic ratio from samples with two copies of CYP2D6, similar to genotyping using allele quantification-based pyrosequencing, as described (Langaee et al., 2015).

### 2.3 CYP2D6 mRNA quantification

The expression levels of CYP2D6 mRNA and the two splice isoforms (CYP2D6∆E3 and CYP2D6∆E6) were measured with real-time qPCR using commercial (Thermo Fisher Scientific) or self-designed TaqMan assays (Zanger et al., 2021) or with SYBR Green using gene-specific primers (primers in Supplementary Table S1), as described (Collins and Wang, 2021a). Multiple commercial or self-designed TaqMan assays were tested; only those that were found to be robust and specific for CYP2D6 were used in this study (Supplementary Table S1). Measurements were conducted on a Quantabio Q real-time PCR instrument (VWR, PA, United States) with *β*-actin as an internal control. Our approach for measuring CYP2D6 mRNA involved three qPCR primer sets. Firstly, the total pool of CYP2D6 mRNA was measured using primers targeting exon 9, found in full-length CYP2D6, CYP2D6∆E3, and CYP2D6∆E6. Secondly, CYP2D6∆E3-isoform-specific primers were designed to measure any CYP2D6 mRNA isoform missing exon 3. Finally, qPCR primers spanning exons 6 and 7 were used to detect any CYP2D6 isoforms containing exon 6 but not CYP2D6∆E6. The amount of the CYP2D6∆E3 isoform was also measured using fragment analysis, as described (Wang et al., 2014).

### 2.4 CYP2D6 protein quantification

Liver lysate preparation (*n* = 189) and CYP2D6 protein quantification using the capillary Western blotting Jess system (ProteinSimple Inc., CA, United States) were conducted as described (Collins and Wang, 2021a). CYP2D6 protein was detected with a rabbit anti-CYP2D6 antibody (1:10, Novus NBP2-67020) followed by an HRP-conjugated anti-rabbit secondary antibody (Biotechne, San Jose, CA, United States). Full-length CYP2D6 protein expression levels were normalized by *β*-actin detected in the same capillary by a mouse anti-β-actin antibody (1:25, Biotechne) followed by a NIR-conjugated anti-mouse secondary antibody (Biotechne, San Jose, CA, United States). The CYP2D6 antibody used in this study is highly specific to human CYP2D6; only a single 53 kD (full-length CYP2D6) or 48 kD (CYP2D6∆E3) was detectable in CYP2D6 cDNA transfected HEK cells, but not in mock-transfected cells nor liver samples with homozygous null allele genotypes (*4/*4 or *5/*3) (Supplementary Table S1). The CYP2D6∆E6 mRNA has a shifted open reading frame and is targeted for nonsense-mediated decay. It is unknown whether CYP2D6∆E6 encodes a protein, although the resulting protein would not be detected by the CYP2D6 antibody used here since the CYP2D6 antibody targets protein residues 301–497 of CYP2D6, which are encoded by 3′portion of exon 6 and the downstream exons, which would be missing from the predicted CYP2D6∆E6 protein (a c-terminal truncated protein of 289 aa) (Wang et al., 2014).

### 2.5 CYP2D6 expression plasmids

Expression plasmids containing CYP2D6 cDNA corresponding to full-length (NM_000106, catalog #SC104446) or CYP2D6∆E3 (NM_001025161, catalog #SC302357) were purchased from Origen (Rockville, MD, United States). The entire CYP2D6 gene (5.1 kb including introns) was PCR amplified from liver gDNA samples and cloned into the pcDNA3 vector using the Infusion cloning kit (Clontech), as described (Wang et al., 2014). Eight clones harboring either rs1065852 G or C alleles (four for each) were selected, representing either the CYP2D6*1 or CYP2D5*10 haplotype, respectively. Haplotypes containing rs16947 were avoided since rs16947 reduces total CPY2D6 expression (Wang et al., 2014), which would have potentially interfered with comparing CYP2D6∆E3 to the total CYP2D6 transcript pool. All constructs were sequenced to ensure sequence fidelity.

### 2.6 Cell culture and transfection

Cells were cultured at 5% CO2/37°C in a humidified incubator in DMEM (HepG2) or DMEM/F12 (HEK293) supplemented with 10% fetal bovine serum, 100 U/mL penicillin, and 100 μg/mL streptomycin. Cells were plated into 12-well plates the day before transfection. Transfection of plasmid DNA was performed using lipofectamine 3,000 (Invitrogen Life Technologies, CA, United States) according to the manufacturer’s instructions. Cells were harvested 48 h after transfection for total RNA preparation or lysed with RIPA buffer for Western blotting. In the CYP2D6 genomic DNA transfection experiments, mRNA was purified from total RNA using the PolyA Tract mRNA isolation system (Promega) to avoid contamination of plasmid DNA. To determine the mRNA stability of the CYP2D6 splice isoforms, HepG2 cells (expressing full-length CYP2D6, CYP2D6∆E3, or CYP2D6∆E6) were treated with actinomycin D (20 μg/mL) for different time periods before harvesting. To test protein stability, CYP2D6, and CYP2D6∆E3 transfected HEK cells were treated with 10 μg/mL cycloheximide for 6 h before harvesting.

### 2.7 Data analysis

After the log10 transformation, the expression levels of all CYP2D6 transcripts followed a normal distribution. *β*-actin normalized full-length CYP2D6 protein levels were normally distributed without transformation. A multiple linear regression model was used to test the association between genotype and the expression levels of the CYP2D6 splice isoforms using the Minitab software (version 21). Forward and backward stepwise regression were used to select the best set of predictors to include in the multiple linear regression models with a cutoff *p*-value < 0.05. The tested candidate predictors were: sex, race, age, CYP2D6 genotypes, and several transcription factors (TFs) known to affect CYP2D6 expression (Collins and Wang, 2021b). To test the association between enhancer SNP rs5758550 or *41 SNP rs28371725 with the overall CYP2D6 mRNA level, race, and the expression levels of several TFs (RXRA, NR1I3, HNF4A, ESR1, FOXA2, and ARNT) were included as covariates. For analysis of the association between rs1058164 and CYP2D6∆E3, age, sex, race, and TFs were not significant, and therefore none were included, while CYP2D6*2 SNP rs16947 and CYP2D6*4 SNP were significant, and were thus included in the model. Age, sex, race, and TFs were not included in the CYP2D6 protein analysis since none of them were significant. A simple linear regression model also tested associations between genotype-predicted expression score (ES) and CYP2D6 protein levels. The goodness-of-fit was judged by residual and normal quartile plots. The Student’s t-test was used to compare the means between two groups and ANOVA for more than two groups.

## 3 Results

### 3.1 CYP2D6 genotype and haplotype frequency in the liver cohort

The allele frequencies of the twelve common CYP2D6 SNPs were similar to those reported in the 1,000 genome database (1000 Genomes Project Consortium et al., 2012) (Supplementary Table S2), with the *10, *6, *9 alleles being more prevalent in EA and *17 and *29 being more prevalent in AA. Gene deletion frequencies were similar between EA and AA (∼5%), while CNVs with >2 copies were more prevalent in AA (20%) than in EA (6%) (Supplementary Table S3). The frequencies of CYP2D6 diplotype in EA and AA are shown in Table 1.

**TABLE 1:** Frequency of CYP2D6 diplotype in liver samples.

Diplotype	AA+ EA		AA		EA	
	Count	%	Count	%	Count	%
*1/*1	25	10.25	5	4.07	20	16.53
*1/*2	42	17.21	19	15.45	23	19.01
*1/*4	13	5.33	4	3.25	9	7.44
*1/*5	3	1.23	3	2.44	0	0.00
*1/*6	1	0.41	1	0.81	0	0.00
*1/*9	1	0.41	1	0.81	0	0.00
*1/*10	5	2.05	2	1.63	3	2.48
*1/*17	12	4.92	10	8.13	2	1.65
*1/*29	5	2.05	4	3.25	1	0.83
*1/*41	11	4.51	8	6.50	3	2.48
*2/*2	12	4.92	4	3.25	8	6.61
*2/*3	11	4.51	2	1.63	9	7.44
*2/*5	3	1.23	2	1.63	1	0.83
*2/*9	1	0.41	0	0.00	1	0.83
*2/*10	6	2.46	4	3.25	2	1.65
*2/*17	3	1.23	2	1.63	1	0.83
*2/*29	5	2.05	3	2.44	2	1.65
*2/*41	7	2.87	2	1.63	5	4.13
*3/*9	1	0.41	0	0.00	1	0.83
*4/*10	3	1.23	1	0.81	2	1.65
*4/*17	6	2.46	0	0.00	6	4.96
*4/*4	2	0.82	0	0.00	2	1.65
*4/*5	1	0.41	0	0.00	1	0.83
*4/*6	1	0.41	0	0.00	1	0.83
*4/*9	5	2.05	5	4.07	0	0.00
*4/*29	5	2.05	5	4.07	0	0.00
*4/*41	1	0.41	1	0.81	0	0.00
*5/*17	1	0.41	1	0.81	0	0.00
*5/*41	2	0.82	1	0.81	1	0.83
*6/*10	1	0.41	0	0.00	1	0.83
*6/*29	1	0.41	1	0.81	0	0.00
*9/*41	1	0.41	0	0.00	1	0.83
*10/*10	1	0.41	0	0.00	1	0.83
*10/*17	3	1.23	2	1.63	1	0.83
*10/*41	2	0.82	0	0.00	2	1.65
*17/*17	2	0.82	1	0.81	1	0.83
*17/*29	3	1.23	3	2.44	0	0.00
*17/*41	1	0.41	1	0.81	0	0.00
*29/*29	3	1.23	2	1.63	1	0.83
*29/*41	1	0.41	1	0.81	0	0.00
*41/*41	3	1.23	1	0.81	2	1.65
*1/*1 × 2	3	1.23	2	1.63	1	0.83
*1/*2 × 2	1	0.41	1	0.81	0	0.00
*1/*4 × 2	3	1.23	3	2.44	0	0.00
*1/*17 × 2	1	0.41	1	0.81	0	0.00
*1/*29 × 2	3	1.23	3	2.44	0	0.00
*2/*4 × 2	2	0.82	1	0.81	1	0.83
*2/*1 × 2	1	0.41	1	0.81	0	0.00
*2 × 2/*2 × 2	2	0.82	2	1.63	0	0.00
*2 × 2/*4 × 3	1	0.41	1	0.81	0	0.00
*4/*1 × 2	1	0.41	1	0.81	0	0.00
*4/*4 × 2	1	0.41	1	0.81	0	0.00
*4/*29 × 2	1	0.41	1	0.81	0	0.00
*9/*29 × 2	1	0.41	0	0.00	1	0.83
*10/*2 × 2	1	0.41	1	0.81	0	0.00
*10/*4 × 2	2	0.82	1	0.81	1	0.83
*29/*2 × 2	1	0.41	0	0.00	1	0.83
*41/*4 × 2	1	0.41	1	0.81	0	0.00
*2 × 2/*4 × 2	1	0.41	0	0.00	1	0.83
*1 × 4/*2 × 5	1	0.41	0	0.00	1	0.83

### 3.2 A commonly-occurring exon 3 SNP rs1058164 is associated with increased expression of a CYP2D6 splice isoform lacking exon 3

**FIGURE 1:**
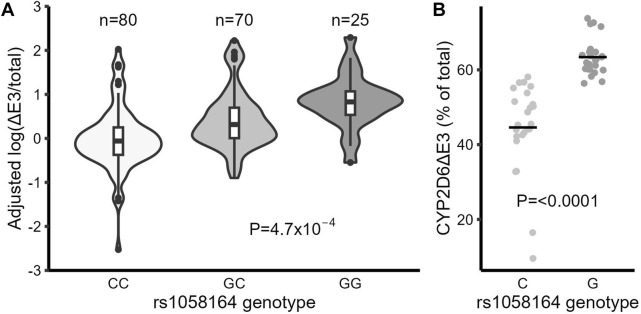
The frequent synonymous SNP rs1058164 G is associated with higher levels of the CYP2D6 splice isoform missing exon 3 (CYP2D6∆E3). **(A)** CYP2D6∆E3 (log) levels in liver samples grouped by rs1058164 genotypes, adjusted by rs16947 and rs3892097 genotypes. The differences between groups were analyzed with multiple linear regression. The box and horizontal lines show the 25th and 75th percentiles surrounding the mean, and the whiskers show values up to 1.5*IQR. **(B)** The expression levels of CYP2D6∆E3 in HEK293 T cells after transfection with CYP2D6 genomic DNA expression plasmids containing rs1085164 G or C alleles. Data are combined results from different clones (4 for each genotype) and two independent experiments with triplicates. The horizontal bar indicates the average (mean).

There are two common alternative splice isoforms for CYP2D6, one lacking exon 6 (CYP2D6∆E6) and another lacking exon 3 (CYP2D6∆E3). A SNP in exon 6 (rs16947, *2) increases the production of CYP2D6∆E6 (Wang et al., 2014). To determine whether any SNPs may also control the expression of CYP2D6∆E3, we used fragment analysis to quantify the fraction of CYP2D6∆E3 isoforms in the total CYP2D6 transcript pool for each sample, as described (Wang et al., 2014). The average fraction of CYP2D6∆E3 in liver samples was 21% (ranging from 1%–48%, with very low levels in CYP2D6*4 carriers), similar to a previous report (Sangar et al., 2010). Using this data, we then tested for association between genotype and the production of CYP2D6∆E3. In a multiple linear regression model that included several SNPs (rs1058164, rs3892097, and rs16947), the most significantly associated SNP with CYP2D6∆E3 was rs1058164 (1662 G >C), with each G allele being associated with 1.48-fold increased CYP2D6∆E3 (*p* = 0.001) (Table 2). To validate these results, we then used real-time qPCR to specifically quantify CYP2D6∆E3. The expression of CYP2D6∆E3 was normalized by total CYP2D6 expression (CYP2D6∆E3/total CYP2D6) to control for overall variable transcription of CYP2D6 between samples. Again, rs1058164 G was associated with 2.5-fold increased CYP2D6∆E3 (*p* = 4.7 × 10^−4^), and the median levels of CYP2D6∆E3 for the various genotype were CC (0.68), CG (1.18) and GG (2.02) (Table 2; Figure 1A), after adjusting for rs3892097 and rs16947. Furthermore, when CYP2D6 genomic DNA is expressed in HEK293 T cells, cells carrying the rs1058164 G allele produced more CYP2D6∆E3 than those transfected with the C allele (*p* < 0.0001) (Figure 1B).

**TABLE 2:** Associations between CYP2D6 genotype and levels of CYP2D6∆E3, E6-containing CYP2D6, and total CYP2D6 mRNA in liver samples using multiple linear regression. Only significant variables were included in the regression models after screening, as described in the method section. The *p*-value for each variable is shown, and the coefficient is in a log10 scale.

SNP ID (independent variable)	Dependent variable	Coefficient	*p*-Value
*Fragment analysis*			
rs1058164 G	log (CYP2D6∆E3)	0.1708	0.001
rs3892097 (*4)	log (CYP2D6∆E3)	−0.2849	0.003
rs16947 (*2)	log (CYP2D6∆E3)	0.1552	0.003
*Real-time qPCR - CYP2D6∆E3*			
rs1058164 G	log (CYP2D6∆E3/total)	0.419	4.7E-4
rs3892097 (*4)	log (CYP2D6∆E3/total)	−0.133	0.187
rs16947 (*2)	log (CYP2D6∆E3/total)	0.332	0.004
*Real-time qPCR - CYP2D6∆E6*			
rs16947 (*2)^a^	Log (CYP2D6 with E6/total)	−0.1379	0.055
rs28371725 (*41)^a^	Log (CYP2D6 with E6/total)	−0.069	0.661
*Real-time qPCR - CYP2D6*			
rs5758550^b^	log (CYP2D6 total)	0.0987	0.009
rs28371725 (*41)^b^	log (CYP2D6 total)	−0.047	0.571

^a^ Simple regression as other screened variables were not found to be significant.

^b^ Adjusted for race and the expression levels of RXRA, NR1I3, HNF4A, ESR1, FOXA2, and ARNT.

We also re-evaluated the effects of the enhancer SNP rs5758550, rs16947 (*2), and rs28371725 (*41) on the expression of CYP2D6 and its splice isoforms. As expected, rs5758550 is associated with increased mRNA expression of total CYP2D6 (1.25-fold, *p* = 0.009, Table 2) after adjusting for the expression of several TFs known to regulate CYP2D6 (Collins and Wang, 2021a). To test the impact of rs16947 on skipping of exon 6, we first attempted to use a custom TaqMan probe that specifically quantifies CYP2D6∆E6 (Zanger et al., 2021), but the assay generated very low amplification efficiency and inconsistent results. Instead, we used a custom exon 6/exon 7-spanning TaqMan assay (Zanger et al., 2021) to specifically quantify CYP2D6 isoforms that contain exon 6. Again, this was normalized by total CYP2D6 expression (CYP2D6 with E6/total CYP2D6). Consistent with our previous results (Wang et al., 2014), rs16947 was associated with decreased expression of CYP2D6 isoforms containing exon 6 (0.87-fold, *p* = 0.055, Table 2), supporting that rs16947 promotes skipping of exon 6, although not quite reaching statistical significance. Conversely, rs28371725 (*41) was not associated with either the overall expression of CYP2D6 or specifically with CYP2D6 isoforms containing exon 6 (Table 2), consistent with our previous study (Wang et al., 2014).

### 3.3 The CYP2D6∆E3 protein is less stable than full-length CYP2D6

**FIGURE 2:**
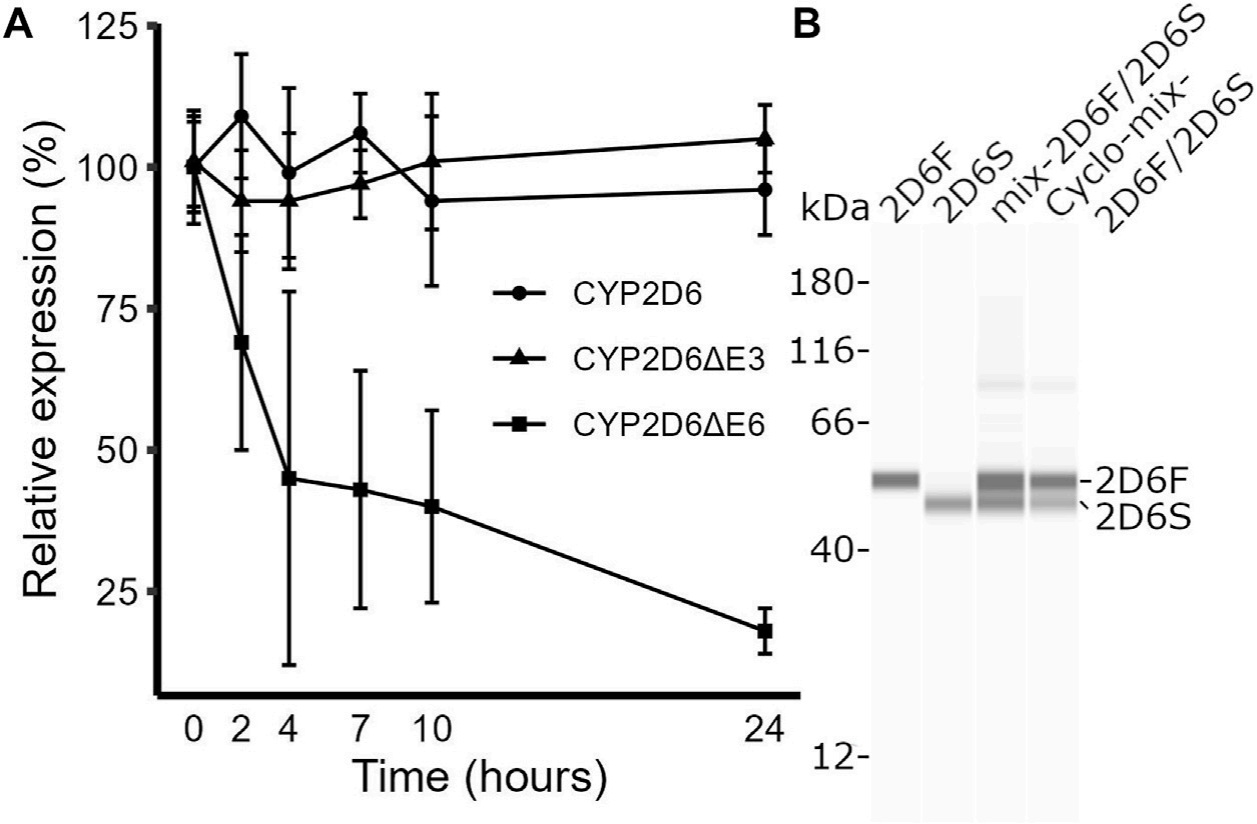
Testing mRNA and protein stabilities of full-length CYP2D6 and its splice isoforms. **(A)** Time course of the relative expression levels of full-length CYP2D6, CYP2D6∆E3, and CYP2D6∆E6 in HepG2 cells after actinomycin D treatment. **(B)** Western blot image of CYP2D6 protein isoforms in HEK293 T cells transfected with plasmid DNAs expressing full-length (HEK2D6F), CYP2D6∆E3 (HEK2D6S), a mix of the two (mix-2D6F/2D6S), or a mix of the two and treated with cycloheximide (Cyclo-mix-2D6F/2D6S).

CYP2D6∆E6 isoforms have a shifted reading frame, causing decreased CYP2D6 mRNA expression due to nonsense-mediated RNA decay (Wang et al., 2014). In contrast, CYP2D6∆E3 isoforms contain an in-frame deletion that lacks 153 bp. We tested whether the CYP2D6∆E3 mRNA is stable by treating HepG2 cells with actinomycin D, which blocks transcription and enables measurement of mRNA longevity. Unlike CYP2D6∆E6, CYP2D6∆E3 was as stable as full-length CYP2D6 (Figure 2A) and therefore did not decrease the total CYP2D6 transcript pool. However, at the protein level, CYP2D6∆E3 appears to be less stable than full-length CYP2D6. In transfected HEK293 T cells, CYP2D6∆E3 protein levels decreased by 53% after treatment with the protein synthesis inhibitor cycloheximide for 6 hours, compared to only a 33% decrease in full-length CYP2D6 (Figure 2B). However, as reported previously (Sangar et al., 2010), we only observed a singular band corresponding to full-length CYP2D6 in liver samples (Supplementary Figure S1), suggesting that the CYP2D6∆E3 protein is highly unstable *in vivo*.

### 3.4 Liver samples with rs1058164 have decreased full-length CYP2D6 protein expression

We next tested for the relationship between the genotype of the aforementioned regulatory variants (rs5758550, rs16947, *41, and rs1058164) and full-length CYP2D6 protein levels in liver samples (*n* = 189) using a simple linear regression model. The overall correlation between total CYP2D6 mRNA and full-length protein is weak (r = 0.356, *p* = 1.11 × 10^−6^), consistent with previous report (Ning et al., 2018). To avoid confounding by CNVs and complex haplotype structure of null alleles, 81 samples carrying CNVs and/or null alleles (*3, *4, *6) were excluded in these analyses, leaving an effective sample size of 108. While *41 was associated with decreased full-length CYP2D6 protein, it did not reach statistical significance (*p* = 0.082), in agreement with its lack of association with total CYP2D6 mRNA levels. Consistent with its effects on total CYP2D6 mRNA expression, rs5758550 was associated with increased full-length CYP2D6 protein (*p* = 1.09 × 10^−12^) (Table 3). rs16947 was also associated with increased full-length CYP2D6 protein in simple linear regression analysis, however, we expected that this was due to its high LD with rs5758550. Indeed, after adjusting for the rs5758550 genotype, rs16947 was instead associated with reduced full-length CYP2D6 protein expression (*p* = 9.14 × 10^−4^) (Table 3), as expected.

**TABLE 3:** Association between CYP2D6 genotype and full-length CYP2D6 protein level in liver samples using simple or multiple linear regression.

SNP ID	Coefficient	*p*-Value
*Simple linear regression*		
rs5758550	65.11	1.09E-12
rs16947	28.8	0.006
rs28371725 (*41)	−25.4	0.082
rs1058164 G	−42.1	5.97E-5
*Multiple linear regression*		
rs1058164 G	−41.8	6.08E-4
rs5758550	72.3	5.66E-12
rs16947 (*2)	−44.5	9.14E-4

**FIGURE 3:**
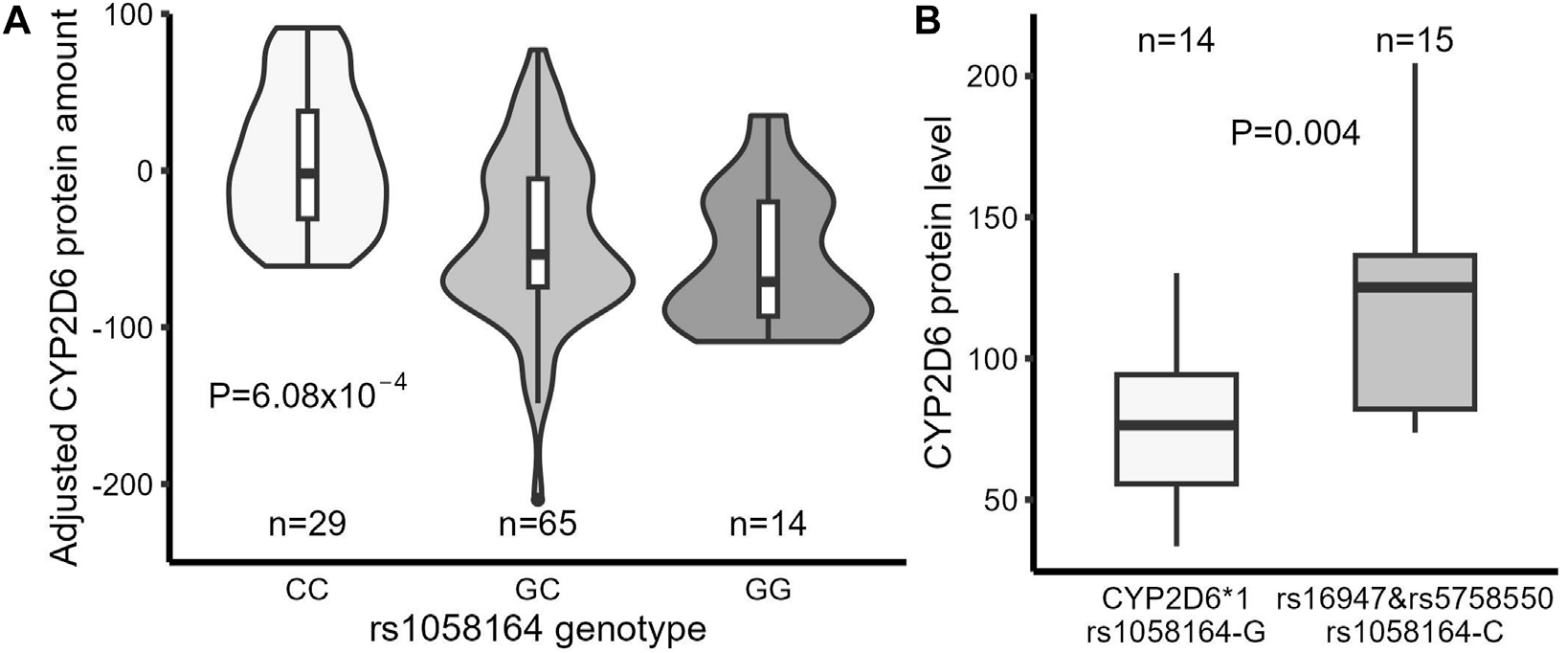
Comparison of full-length CYP2D6 protein expression in liver samples between different genotypes. **(A)** Full-length CYP2D6 protein levels in liver samples (adjusted by rs5758550 and rs16947 genotypes) grouped by rs1058164 genotype. **(B)** Full-length CYP2D6 protein expression in CYP2D6*1 samples (carrying rs1058164 G) and CYP2D6*2 samples (carrying rs16947, rs1058164 C, and rs5758550). Only samples with a single copy of a functional CYP2D6 gene were analyzed. In both plots, the box and horizontal lines show the 25th and 75th percentiles surrounding the mean, and the whiskers show values up to 1.5*IQR.

The rs1058164 G allele was associated with decreased full-length CYP2D6 protein level in simple linear regression analysis (*p* = 5.97 × 10^−5^). A similar association (*p* = 6.08 × 10^−4^) was seen in a multiple linear regression analysis after adjusting for rs5758550 and rs16947 (Figure 3A; Table 3), with each G allele being associated with a ∼50% reduction in full-length CYP2D6 protein. Notably, since the CYP2D6*1 haplotype contains rs1058164 G and most of the CYP2D6*2 haplotypes contain rs1058164 C, the CYP2D6*1 haplotype would be expected to have lower protein than *2 haplotypes that also contain rs5758550. To test this, we specifically looked at protein expression in samples with only one functional copy of CYP2D6 (i.e., samples were *1/*null or *2/*null) so that full-length CYP2D6 protein expression would be solely driven by either a single *1 or *2 allele. In this subset (*n* = 29), samples with CYP2D6*1 had significantly lower full-length CYP2D6 protein levels than samples with rs16947 and rs5758550 (78 ± 28 vs. 120 ± 42, *p* = 0.004) (Figure 3B).

### 3.5 The impact of CNVs on CYP2D6 protein expression

**FIGURE 4:**
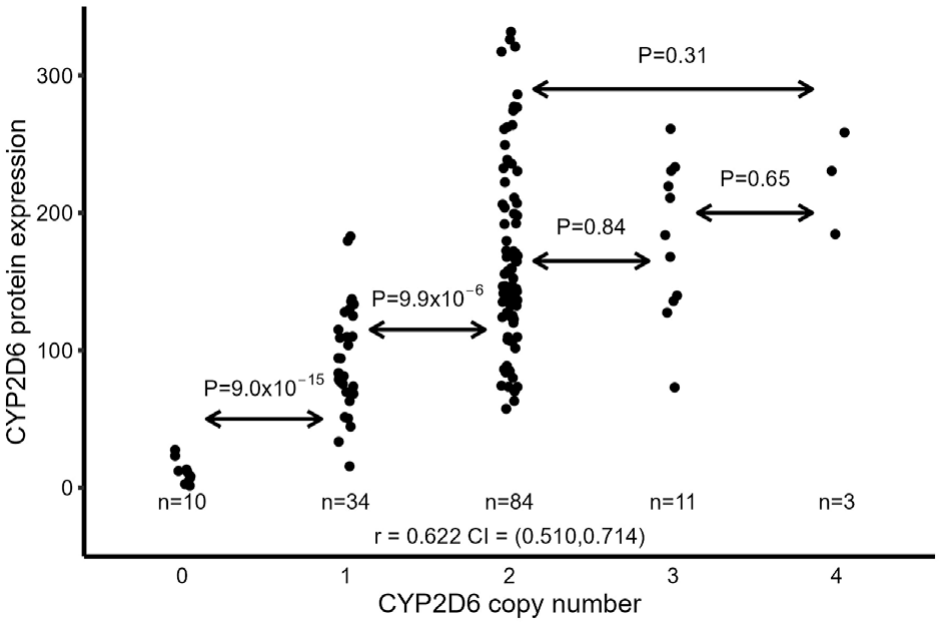
Relationship between CYP2D6 CNV and full-length CYP2D6 protein expression in liver samples. Samples with duplicated null alleles (*3, *4, *6) and *41 CNV >4 were excluded.

According to the accepted standards, CYP2D6 gene duplication is thought to double CYP2D6 expression and activity if the duplicated genes are either *1 or *2 (Crews et al., 2014; Hicks et al., 2017). However, duplication of the CYP2D6 gene does not include duplication of the distal enhancer located >100 kb downstream of the CYP2D6 promoter, which is critical for CYP2D6 expression (Wang et al., 2014). Therefore, the duplicated CYP2D6 gene may not be fully expressed. We then tested the relationship between CYP2D6 CNVs and full-length CYP2D6 protein expression in the liver samples. Samples were excluded if they had duplicated null alleles (*3, *4, *6) and *41 (considered to be a reduced expression allele (Crews et al., 2014; Hicks et al., 2017)) or if copy number >4 (due to difficulties in assigning haplotypes for each copy). Samples with duplication of amino acid-changing alleles (*9, *10, *17, and *29) were included since there is no direct evidence showing that these SNPs alter full-length CYP2D6 protein expression. Overall, there is a good correlation between CYP2D6 CNV and full-length CYP2D6 protein (r = 0.622, *p* = 1.0 × 10^−16^) (Figure 4). However, the correlation is mainly driven by samples with CNV ≤ 2 (0, 1 and 2), as the correlation was relatively unchanged (r = 0.646, *p* = 2.0 × 10^−16^) after omission of samples with CNV 3 or 4 and the correlation disappeared (r = 0.165, *p* = 0.105) when samples with CNV 0 and 1 were removed. Also, the comparison of mean CYP2D6 protein levels between CNV groups showed a significant increase between 0 vs. 1 copies and 1 vs. 2 copies, but not with additional CNVs: 2 vs. 3 (*p* = 0.84), 3 vs. 4 (*p* = 0.65), and 2 vs. 4 (*p* = 0.31) (Figure 4).

### 3.6 A model incorporating all three regulatory variants better predicts CYP2D6 protein expression

We then generated a modified version of the AS system (Gaedigk et al., 2008) to predict the full-length CYP2D6 protein expression of each haplotype based on the combined effects of all regulatory variants and CNVs, which we labeled as an expression score (ES) system. We tested three different models for ES assignments (Supplementary Table S4).

Model 1 was based on the accepted standard (Hicks et al., 2017), with ES = 0 for null alleles (*3, *4, *5, *6), ES = 0.5 for the reduced expression allele *41, and ES = 1 for *1, *2, and *39. Star alleles with nonsynonymous changes (*9, *10, *17, and *29) were also assigned ES = 1 as there is no evidence that these SNPs change the expression of full-length CYP2D6 protein. For CNV, ES = (number of CYP2D6 copies) × (ES of corresponding alleles).

Model 2 is a modified version of model 1 that also includes rs5758550 and rs16947 and is based on previous reports (Wang et al., 2014; Ray et al., 2019). Haplotypes with both rs5758550 and rs16947 were assigned ES = 1, haplotypes containing only rs16947 and not rs5758550 were assigned ES = 0.5, and haplotypes containing rs5758550 without rs16947 were assigned ES = 2. This alteration to model 1 applies to all rs16947-containing alleles, such as *17, *29, and *41. For CNV, each additional normal allele only increases the ES by 1.5 instead of two, as in model 1. For example, duplication of normally expressing alleles (e.g., *1) was assigned ES = 1.5 (instead of 2), and duplication of a reduced expression allele (i.e., rs16947 without rs5758550) had an ES = 0.75 (ES = 0.5 + 0.25). Model 3 further expanded on model 2 to also include rs1058164, with a 50% reduction in ES score for each rs1058164 G allele.

**FIGURE 5:**
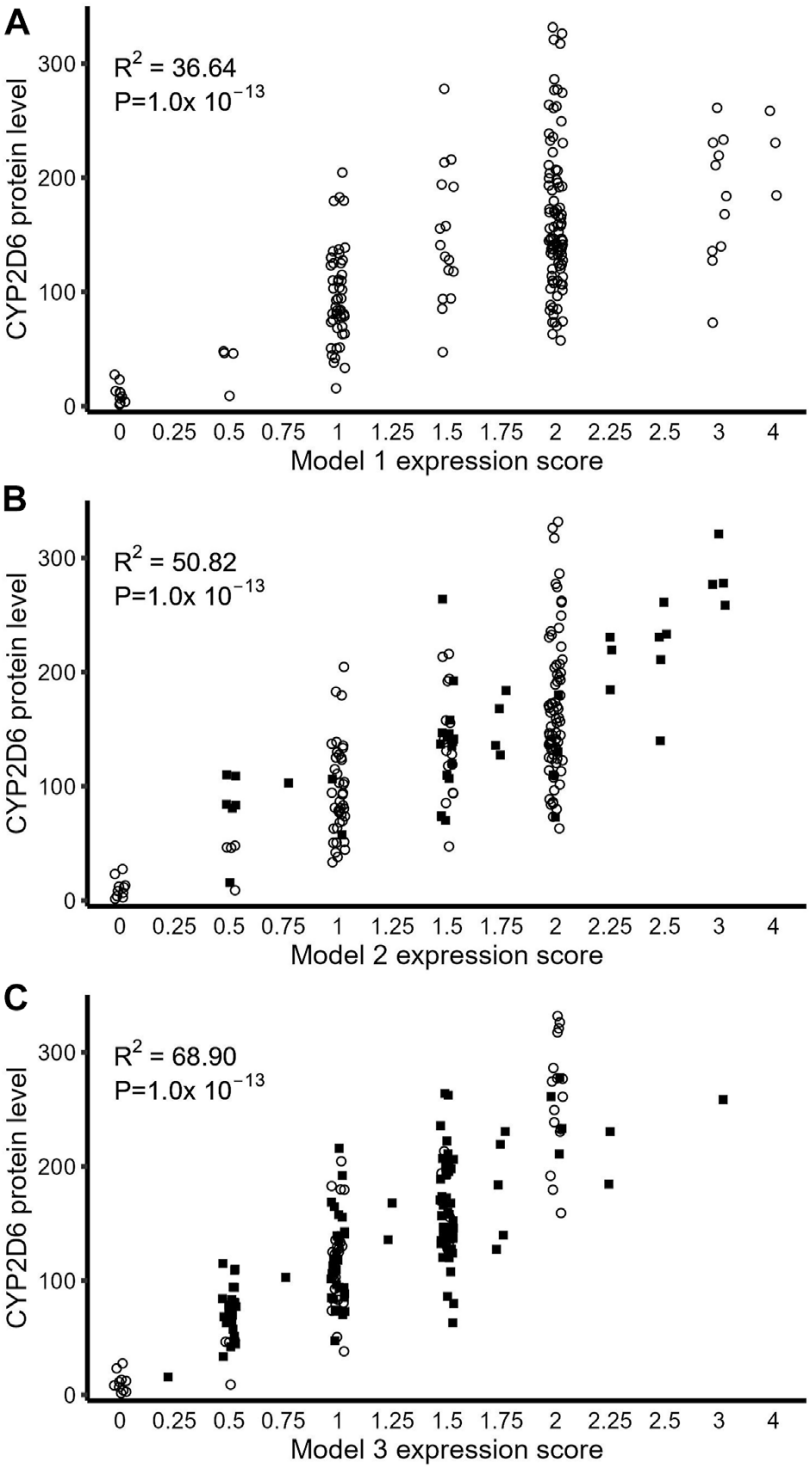
Relationship between full-length CYP2D6 protein expression and expression score derived from genotype. **(A)** CYP2D6 protein level as predicted by ES using Model 1. **(B)** ES-based prediction of CYP2D6 protein *via* model 2. **(C)** Model 3 ES-based prediction of CYP2D6 protein. In all three plots, open circles represent samples that did not move ES values compared to model 1, while closed squares are samples which moved to new ES values based on the particular model.

As shown in both Table 4 and Figure 5, in all three models, ES was significantly associated with measured full-length CYP2D6 protein level (*p* < 10^–13^). Consistent with previous reports (Wang et al., 2014; Ray et al., 2019), the addition of rs16947 and rs575850 in model 2 improved the linearized relationship between ES and CYP2D6 protein level and explained 14.18% more variability in full-length CYP2D6 protein expression than did model 1. Model 3, with its inclusion of rs1058164, had the best-linearized relationship between ES and measured full-length CYP2D6 protein and could further explain an additional 17.27% variability in full-length CYP2D6 protein expression (R^2^ = 68.09%). Model 3 also performed best when the two populations were analyzed separately (Table 4). Notably, improvements from model 1 to model 2 were greater for AA than EA (differences in R^2^, 22.5% vs. 6.55%), while the improvement from model 2 to model 3 was greater in EA than AA (differences in R^2^, 27.58% vs. 3.73%).

**TABLE 4:** Association between full-length CYP2D6 protein level and predicted expression scores (ES) based on the three models using simple linear regression.

Model	AA+ EA			AA only			EA only		
	Coefficient	*p*-value	*R* ^2^ (%)	Coefficient	*p*-value	*R* ^2^ (%)	Coefficient	*p*-value	*R* ^2^ (%)
Model 1	57.94	< 10^−13^	36.64	55.61	1E-11	40.08	62.75	4.27E-11	35.62
Model 2	77.44	< 10^−13^	50.82	81.61	< 10^−13^	62.58	72.82	2.15E-13	42.17
Model 3	106.15	< 10^−13^	68.09	98.63	< 10^−13^	66.31	112.78	< 10^−13^	69.75

## 4 Discussion

We identified a frequent synonymous SNP rs1058164 as a novel regulator for a CYP2D6 alternative splice isoform CYP2D6∆E3. Our results showed that the rs1058164 G allele was associated with increased CYP2D6∆E3 production and decreased full-length CYP2D6 protein expression. Since all CYP2D6*1 haplotypes contain rs1058164 G, this result indicates that the expression or activity of CYP2D6*1 may currently be over-estimated. Using a liver sample cohort, we also re-evaluated the effects of the enhancer SNP rs5758550 and the *2 core SNP rs16947 on the expression of CYP2D6 mRNA and protein levels and found results consistent with our previous report (Wang et al., 2014). Moreover, using an ES system to predict full-length CYP2D6 protein expression from genotypes, we demonstrated that two ES-modified models (model 2 and model 3) that further incorporate CYP2D6 regulatory SNPs better predicted full-length CYP2D6 protein expression than the currently accepted standard (model 1) (Crews et al., 2014; Hicks et al., 2017). Taken together, our results indicate that various 3-SNP-haplotypes (formed by rs5758550, rs16947, and rs1058164) explain a significant portion of inter-person variability in the expression of CYP2D6 protein.

Alternative splicing is an essential mechanism in the regulation of gene expression and protein diversity across eukaryotes. Splicing is regulated by a complex network of trans-acting factors and cis-acting genetic elements (Wang et al., 2015). It has been suggested that genetic alterations to splicing are one of the primary mechanisms underlying genetic associations (GTEx Consortium, 2020), and the most prevalent type of alternative splicing arising from genetic variation is exon skipping, with the majority of the causal SNPs occurring within the skipped exons (Amoah et al., 2021). The two CYP2D6 SNPs (rs16947 and rs1058164) described in this report are also located within their respective exons and may potentially be driving the exon skipping. Embedded within CYP2D6*1, rs1058164 G is a minor allele with a frequency ranging from 0.27 to 0.43 in different populations, while the major allele C is on >70 star alleles (PharmVar), including several common ones (*10, *17, *29). With the exception of one *2 sub-haplotype (CYP2D6*2.004), most of the rs16947-variant T containing alleles also have rs1058164 C (PharmVar), indicating that rs1058164 G and rs16947 T rarely co-occur. Indeed, we only found seven rs16947 T (3% of all rs16947 containing alleles) haplotypes that also had rs1058164 G in our liver samples. Since skipping of both exon 3 and exon 6 reduced the expression of full-length CYP2D6 protein, it is possible that the apparent mutual exclusivity of rs16947 T and rs1058164 G may be due to a need to balance the expression/function of CYP2D6 proteins.

Based on protein structure (Rowland et al., 2006), the CYP2D6∆E3 isoform lacks numerous residues that form the active site cavity (residues 118–122) or are involved in heme binding (Trp-128 and Arg-132), likely causing it to have severely reduced or no enzymatic activity. While CYP2D6∆E3 has previously been detected at the mRNA level by us and others (Zhuge and Yu, 2004; Sangar et al., 2010; Wang et al., 2014), to our knowledge, it has not been detected at the protein level *in vivo*. As CYP enzymes form protein complexes that can directly (Reed et al., 2012) or indirectly (Kandel and Lampe, 2014) impact the enzymatic activity of other CYPs, it is possible that CYP2D6∆E3 isoforms are actively targeted for degradation to limit their potential deleterious effect on the other CYP enzymes.

Although considered as a reference or wildtype allele, our results indicated that CYP2D6*1 harbors a variant associated with reduced full-length CYP2D6 protein expression, below even CYP2D6*2 alleles containing the enhancer SNP rs5758550 (Figure 3B). These results are consistent with a previous report showing lower CYP2D6 protein levels in liver samples carrying *1 than *2A, a *2 allele with a promoter SNP rs1080985 (that is in high LD with rs5758550) (Zanger et al., 2001). Our results also agree with a recent GWAS study showing that rs1058164 C is associated with increased levels of CYP2D6 protein in liver samples and CYP2D6-dependent endoxifen (Khor et al., 2023). In our liver cohort, the frequency of the CYP2D6*1 haplotype was 31%, with 10% of the samples being homozygous for CYP2D6*1. Therefore, our results indicate that CYP2D6 metabolic phenotype assignment may be overestimated for a significant portion of individuals. Large variability has been observed in CYP2D6 phenotype groups based on the AS system (Crews et al., 2014; Hicks et al., 2017), which may in part be driven by both *2 and *1 being assigned a normal AS of 1, instead of *1 being considered a reduced expression allele. However, *in vitro* and population pharmacokinetic studies have shown that the amino acid change (R296C) caused by rs16947 in CYP2D6*2 reduces enzymatic activity for some substrates (Abduljalil et al., 2010; Muroi et al., 2014; Hertz et al., 2015; van der Lee et al., 2021; Frederiksen et al., 2022) and further separation of CYP2D6*1 and CYP2D6*2 phenotypes may be necessary for different substrates.

We re-evaluated the impacts of the enhancer SNP rs5758550 and rs16947 on the expression of CYP2D6 mRNA, splice isoforms, and protein in liver samples. Our results showed strong effects of rs5758550 and rs16947 on full-length CYP2D6 protein expression, which, together with rs1058164, explained 45% (Table 3) of the variability in full-length CYP2D6 protein abundance in the liver samples. These results are consistent with previous reports showing that including rs5758550 and rs16947 into a new AS system better predicts CYP2D6 activity (Wang et al., 2014; Ray et al., 2019; Thomas et al., 2020). However, there have been a few studies that debate the influence of rs5758550 and rs16947. For example, rs5758550 apparently does not alter CYP2D6*2 activity towards the metabolism of tamoxifen (Sanchez-Spitman et al., 2017) or dextromethorphan (Dinh et al., 2022) but does impact atomoxetine metabolism (Dinh et al., 2022). These discrepancies may be due to a variety of reasons, including the highly polymorphic nature of CYP2D6, the small sample sizes used in the studies, the analysis methods used, and the capacity of a substrate drug to serve as a surrogate for CYPD6-specific enzyme activity and/or protein abundance. In support of our results, a recent report with a relatively large sample size (n = 497) showed that distal regulatory variants were associated with CYP2D6-dependent metabolism of tamoxifen (independent of CYP2D6 variants in the AS system), and rs5758550 was found to compensate for the deleterious effects of rs16947 (Khor et al., 2023).

We did not find an association between rs28371725 (core SNP of *41) and CYP2D6 mRNA, CYP2D6∆E6 isoform production, or full-length CYP2D6 protein expression (Table 2; Table 3), which agrees with our previous results (Wang et al., 2014). However, there are multiple studies showing reduced CYP2D6 expression/activity in *41 (Abduljalil et al., 2010; van der Lee et al., 2021; Khor et al., 2023), which we hypothesize is due to rs28371725 acting as a surrogate marker for the haplotypes comprised of rs16947 without rs5758550 (i.e., H3-haplotype described in (Ray et al., 2019)). Unlike the other reports (Abduljalil et al., 2010; van der Lee et al., 2021; Khor et al., 2023), our liver cohort consisted of ∼50% AA samples where the concordance between *41 and H3 is the lowest (Ray et al., 2019). In our cohort, all *41 alleles were found in H3-haplotypes in EA, whereas only 81% of the *41 alleles were on H3-haplotypes in AA. Also, while only 6.5% of the H3-haplotypes in EA samples did not have rs28371725 (*41), 18.1% of AA H3-haplotypes did not (allele frequencies were consistent with (Ray et al., 2019)). Therefore, this LD breakage of rs28371725 (*41) and the H3-haplotypes in our cohort enabled us to dissect the roles of rs28371725, rs16947, and rs5758550 on full-length CYP2D6 protein expression and our results did not support an independent effect of rs28371725 on CYP2D6 transcripts and protein expression.

Moreover, the molecular mechanism underlying how rs28371725 (*41) impacts CYP2D6 expression is still unclear; it is an intronic variant that was proposed to cause partial intron 6 retention (Rau et al., 2006) and skipping of exon 6 (Toscano et al., 2006). Recently, a tri-allelic haplotype (consisting of rs16947, *41, and a SNP in exon 9 rs1135840 C) was reported to cause CYP2D6∆E6 in Huh-7 and COS-7 transfection experiments (Zanger et al., 2021). These results disagree with our previous study showing no differences in CYP2D6∆E6 levels between *2 and *41 in either liver samples or in transfected HEK293 T cells (Wang et al., 2014). Unlike *41, the rs1135840 C variant always co-occurs with *2 and is in high LD with rs1058164 C. It is unclear how a nucleotide substitution in exon 9 could affect the skipping of exon 6. Also, as Huh-7 cells express detectable levels of CYP2D6 (Ct value ∼26 indicating moderate expression), natively-expressed CYP2D6 may have confounded their quantification of transfected CYP2D6. Moreover, many of the rare haplotypes (for example, rs16947 T-rs1058164G) presented in (Zanger et al., 2021) were generated by site-directed mutagenesis with *1 as the template. While it is unclear whether rs1058164 G (found on *1) can affect CYP2D6∆E6 production, our results presented here indicated that CYP2D6∆E3 (produced by rs1058164 G) and CYP2D6∆E6 may be mutually exclusive.

Our results also indicated that CYP2D6 expression may be over-estimated in individuals with gene duplication/multiplications since we found no linear relationship between CYP2D6 expression and CNV number in samples carrying CNV >2. Several studies have shown that CYP2D6 activity overlaps between UM (gene duplication being the main cause) and EM phenotypes (Hertz et al., 2015; de Andres et al., 2021; van der Lee et al., 2021), which may arise from an overestimation of the impact of CYP2D6 CNVs. Previously, we found that deletion of the downstream enhancer reduced CYP2D6 expression by half (Wang et al., 2014), and as enhancer-mediated regulation of every CYP2D6 copy may not be equal and additional duplicated genes may be under-expressed compared to a singular copy. As enhancer-mediated regulation is complex, future experiments to determine the role of the downstream enhancer in regulating multiple CYP2D6 gene copies are warranted.

While the new models made significant improvements for predicting CYP2D6 protein expression, we observed differences in how they performed for the two populations. Model 2 was more robust for predicting CYP2D6 protein levels in AA, while model 3 made a larger difference for predicting EA protein (Table 4). This is likely due to population differences in the distribution and LD of the three regulatory SNPs and CYP2D6 CNV frequencies. For example, in the EA population, most of the rs16947-containing haplotypes also contained rs5758550, and in model 2, they were assigned an ES of 1, which is the same value they received in model 1. However, in AA, the concordance between rs16947 and rs5758550 was low, and therefore the ES for many rs16947-containing alleles was misassigned in model 1. Also, AA samples had a higher frequency of >2 CYP2D6 CNVs than EA. Since model 2 specifically corrected misassigned rs16947 alleles and CNVs, it makes sense that it made such a large improvement for the prediction of full-length CYP2D6 protein abundance in AA. In contrast, model 3 addressed the contribution of rs1058164 G alternative splicing on the CYP2D6*1 allele, which is more frequent in EA than in AA, and likely drives the better improvement of model 3 for EA. Many pharmacogenomic panels show differences in their ability to predict phenotypes for diverse populations (Ortega and Meyers, 2014), and our results echo the need for more research on diverse populations to help better design panels for use in personalized drug therapy.

A limitation of this study is that only common SNPs were genotyped, and rare SNPs that may affect full-length CYP2D6 protein expression (e.g., *11, *15, *19, *etc.*) were not captured. However, although these rare alleles can potentially impact CYP2D6 protein expression in individuals, their impact on variability at a population level is expected to be limited. We measured CYP2D6 protein levels instead of activity since we focused on testing the effect of regulatory variants on CYP2D6 mRNA and protein expression. However, although we cannot fully account for the extent that the CYP2D6 regulatory variants influence *in vivo* CYP2D6 activity, their impact may be large as CYP2D6 protein level is the major contributor to interindividual variability in CYP2D6-mediated drug metabolism (Ning et al., 2018). Considering the improvements made by model 3 to predicting full-length CYP2D6 expression, additional studies to evaluate the impact of these regulatory SNPs on CYP2D6 activity and their applicability to additional populations are warranted.

## Data Availability

The original contributions presented in the study are included in the article/Supplementary Materials, further inquiries can be directed to the corresponding author.

## Abbreviations

AA; African American, AS; activity score, CNV; gene copy number variation, CPIC; Clinical Pharmacogenomics Implementation Consortium, CYP; cytochrome P450, EA; European American, ES; expression score, TF; transcription factor, *−; star allele.
