# Supplementary material for: A frequent CYP2D6 variant promotes skipping of exon 3 and reduces CYP2D6 protein expression in human liver samples
Source: Front Pharmacol. 2023 Jul 27;14:1186540. doi: 10.3389/fphar.2023.1186540 (PMC10412816; doi:10.3389/fphar.2023.1186540)
Supplement: Supplementary file 1 [file DataSheet1.PDF]

Table S1. Sequences of PCR and Snapshot primers and TaqMan assay ID

**CYP2D6 genotyping**	
**PCR primers**	**Sequence**
enhancer-F	TGATTCTGACAGGTTTTCTTTTGC
enhancer-R	GAATTCCTAAAATAGTCCTGATGCAAG
2D6-long-F	CCAGAAGGCTTTGCAGGCTTCA
2D6-long-R	ACTGAGCCCTGGGAGGTAGGTA
**Snapshot primers**	**sequence**
100C>T (*10, rs1065852)	ACGCTGGGCTGCACGCTAC
1022 C>T (*17, rs28371706)	(T3) ACCGCCCGCCTGTGCCCATCA
1662 G>C (rs1058164)	(T11) CAAGTTGCGCAAGGTGGA
1708 delT (*6, rs5030655)	(T14)GCAAGAAGTCGCTGGAGCAG
1847 G>A (*4, rs3892097)	(T21)CCGCATCTCCCACCCCCA
2550 delA (*3, rs35742686)	(T23)GATGAGCTGCTAACTGAGCAC
2616 delAAG (*9, rs5030656)	(T28)GCCTTCCTGGCAGAGATGGAG
2851 C>T (*2, rs16947)	(T33)AGCTTCAATGATGAGAACCTG
4181 G>C (*39, rs1135840)	(T38)GTGTCTTTGCTTTCCTGGTGA
2989 G>A (*41, rs28371725)	(T45)AGTGCAGGGGCCGAGGGAG
3184 G>A (*29, rs59421388)	(T47)GTCCAACAGGAGATCGACGAC
enhancer SNP A>G (rs5758550)	(T48) TTAAAGATTCCCATTCCACAGTTTTTT
**CYP2D6 specific primers for mRNA amplification**	
2D6mRNAF	CCCATTTGGTAGTGAGGCAGGT
2D6mRNAR	ACCAGGAAAGCAAAGACACCATG
**Fragment analysis primers**	
2D6E2F-FAM	ACGTGTTCAGCCTGCAGCTG
2D6E4R	TTGTCCAAGAGACCGTTGG
**Quantitative Real-time PCR**	**Sequence**
total CYP2D6, forward	TGTGAAGCCGGAGGCCT
total CYP2D6, reverse	CAGGAAAGCAAAGACACCATGG
b-actin-F	GAGAAGAGCTACGAGCTGCCT
b-actin-R	GGTAGTTTCGTGGATGCCAC
**TaqMan assays, quantification**	**TaqMan probes**
CYP2D6ΔE3	Hs02576167-m1, used in this study
CYP2D6 spaning E4-E5	Hs02576168-g1, not specific to CYP2D6, not used in this study
CYP2D6 spaning E3-E4	Hs00164385_m1, probe contains a SNP confirmed by the manufacturer, not used in this study
CYP2D6 spaning E6-E7	self-designed as reported, used in this study
CYP2D6 sapning E5-E7	self-designed as reported, low amplification efficiency, not used in this study
**TaqMan assays, CNV**	**TaqMan probes**
CYP2D6 CNV-exon9	Hs00010001-cn
CYP2D6 CNV-int6	Hs04502391-cn

Table S2. Allele frequency of common SNPs of CYP2D6 in liver samples

	Variant Allele Frequency					
	Current Study			1000 genome		
SNP ID	AA + EA	AA	EA	Global	African	European
100C>T (*10, rs1065852)	0.194	0.162	0.227	0.211	0.136	0.218
1022 C>T (*17, rs28371706)	0.065	0.11	0.024	0.013	0.089	0.002
1662 G>C (rs1058164)	0.621	0.621	0.619	0.576	0.634	0.576
1708 delT (*6, rs5030655)	0.008	0.008	0.028	0.002	0.002	0.001
1847 G>A (*4, rs3892097)	0.141	0.121	0.161	0.182	0.093	0.191
2550 delA (*3, rs35742686)	0.004	0.004	0.004	0.011	0.004	0.013
2616 delAAG (*9, rs5030656)	0.014	0.004	0.024	0.018	0.006	0.022
2851 C>T (*2, rs16947)	0.456	0.495	0.417	0.321	0.339	0.318
4181 G>C (*39, rs1135840)	0.622	0.617	0.628	0.573	0.62	0.567
2989 G>A (*41, rs28371725)	0.071	0.065	0.078	0.091	0.038	0.104
3184 G>A (*29, rs59421388)	0.077	0.113	0.041	0.0004	0.083	0.0004
enhancer SNP A>G (rs5758550)	0.322	0.321	0.324	0.26	0.383	0.247

Table S3. Frequency of copy number variations in liver samples

	AA + EA		AA		EA	
CNV Copy	Count	%	Count	%	Count	%
1	13	5.33	7	5.69	6	4.96
2	198	81.15	91	73.98	108	89.30
3	25	10.25	20	16.26	5	4.13
4	5	2.05	4	3.25	1	0.83
5	2	0.82	1	0.81	1	0.83

Table S4. Expression score (ES) for each diplotype based on three models

	Expression Score (ES)		
Diplotype	Model 1 (ES1)	Model 2 (ES2)	Model 3 (ES3)
*1/*1	2	2[a]	ES2-1[d]
*1/*2	2	1.5 or 2[b]	ES2-0.5[e]
*1/*4	1	1	ES2-0.5
*1/*5	1	1	ES2-0.5
*1/*6	1	1	ES2-0.5
*1/*9	2	2	ES2-1[d]
*1/*10	2	2	ES2-0.5
*1/*17	2	1.5 or 2[b]	ES2-0.5
*1/*29	2	1.5 or 2[b]	ES2-0.5
*1/*41	1.5	1.5 or 2[b]	ES2-0.5
*2/*2	2	1, 1.5 or 2[b]	Same as ES2
*2/*3	1	0.5 or 1[b]	Same as ES2
*2/*5	1	0.5 or 1[b]	Same as ES2
*2/*9	2	1.5 or 2[b]	ES2-0.5[d]
*2/*10	2	1.5 or 2[b]	Same as ES2
*2/*17	2	1, 1.5 or 2[b]	Same as ES2
*2/*29	2	1, 1.5 or 2[b]	Same as ES2
*2/*41	1.5	1, 1.5 or 2[b]	Same as ES2
*3/*9	1	1	ES2 -0.5
*4/*10	1	1	Same as ES2
*4/*17	1	0.5 or 1[b]	Same as ES2
*4/*4	0	0	Same as ES2
*4/*5	0	0	Same as ES2
*4/*6	0	0	Same as ES2
*4/*9	1	1	ES2 -0.5
*4/*29	1	0.5 or 1[b]	Same as ES2
*4/*41	0.5	0.5 or 1[b]	Same as ES2
*5/*17	1	0.5 or 1[b]	Same as ES2
*5/*41	0.5	0.5 or 1[b]	Same as ES2
*6/*10	1	1	Same as ES2
*6/*29	1	0.5 or 1[b]	Same as ES2
*9/*41	1.5	1.5 or 2[b]	Same as ES2
*10/*10	2	2	Same as ES2
*10/*17	2	1.5 or 2[b]	Same as ES2
*10/*41	1.5	1.5 or 2[b]	Same as ES2
*17/*17	2	1, 1.5 or 2[b]	Same as ES2
*17/*29	2	1, 1.5 or 2[b]	Same as ES2
*17/*41	1.5	1, 1.5 or 2[b]	Same as ES2
*29/*29	2	1, 1.5 or 2[b]	Same as ES2
*29/*41	1.5	1, 1.5 or 2[b]	Same as ES2
*41/*41	1	1, 1.5 or 2[b]	Same as ES2
*1/*1x2	3	2.5[c]	ES2 -[1*0.5 + 1.5*0.5]
*1/*2x2	3	1+ (0.5 or 1)*1.5[b,c]	ES2 - 0.5
*1/*4x2	1	1	ES2 - 0.5
*1/*17x2	3	1+ (0.5 or 1)*1.5[b,c]	ES2 - 0.5

*1/*29x2	3	1+ (0.5 or 1)*1.5[b,c]	ES2 - 0.5
*2/*4x2	1	0.5 or 1[b]	Same as ES2
*2/*1x2	3	(0.5 or 1) + 1.5[b,c]	ES2 -[1.5*0.5]
*2x2/*2x2	4	[(0.5 or 1)*1.5] *2[b,c]	Same as ES2
*2x2/*4x3	2	(0.5 or 1) *1.5[b,c]	Same as ES2
*4/*1x2	2	1.5[c]	ES2 -[1.5*0.5]
*4/*4x2	0	0	Same as ES2
*4/*29x2	2	(0.5 or 1)*1.5[b,c]	Same as ES2
*9/*29x2	3	1+ (0.5 or 1)*1.5[b,c]	ES2 - 0.5[d]
*10/*2x2	3	1+ (0.5 or 1)*1.5[b,c]	Same as ES2
*10/*4x2	1	1	Same as ES2
*29/*2x2	3	1+ (0.5 or 1)*1.5[b,c]	Same as ES2
*41/*4x2	0.5	0.5 or 1[b]	Same as ES2
*2x2/*4x2	2	(0.5 or 1) *1.5[b,c]	Same as ES2
*1x4/*2x5	5	Unknown	Unknown

a. ES=1 per *1 allele if rs5758550 is absent; ES=2 per *1 allele if rs5758550 is present

b. ES=0.5 if rs16947 is present and rs5758550 is absent; ES=1 if both rs16947 and rs5758550 are present

c. Duplicated genes do not have enhancer, reducing expression to 50%. Thus, the ES for *1x2 is 1.5

d. *1 and *9 haplotypes contain rs1058164 G allele, thus ES3 for *1 or *9 per allele equals [ES2*0.5=0.5]

e. Most of rs16947 haplotypes contain rs1058164 C, while some with rs1058164 G (i.e. CYP2D6*2.004 haplotype) If contain rs1058164 G, ES3=ES2*0.5

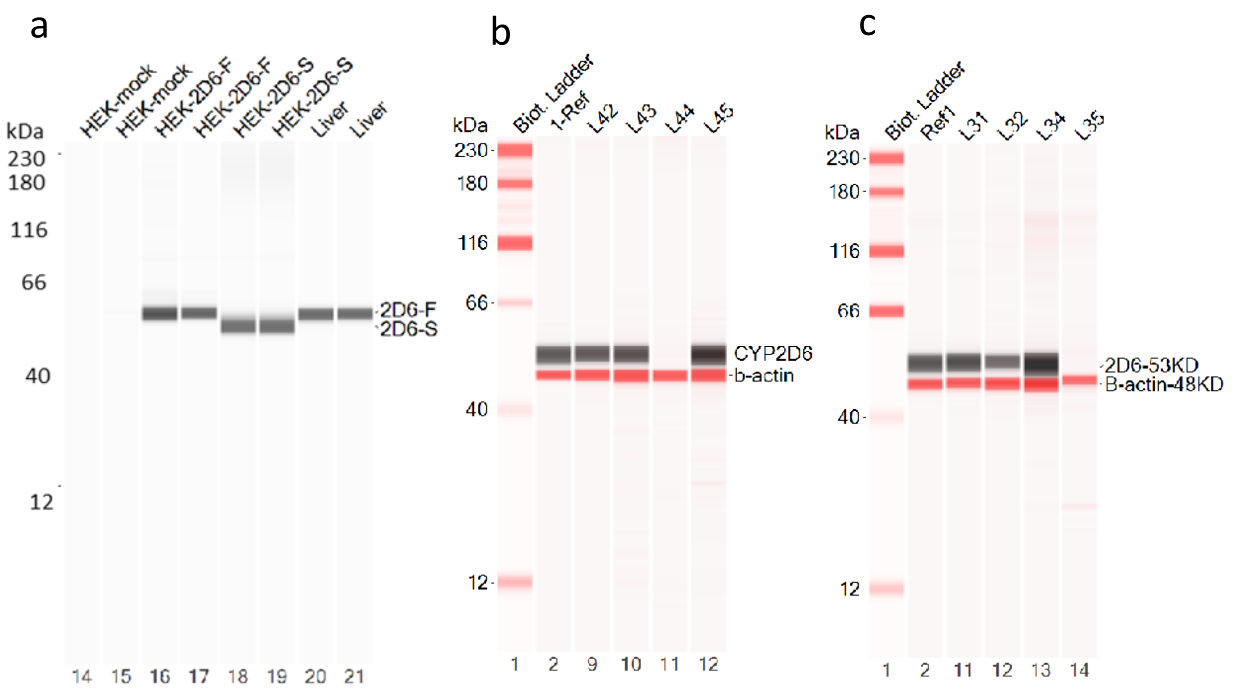

Supplemental Figure S1. Western blot images of CYP2D6 protein. Lysates (0.5-1 µg protein) from transfected HEK cells (a) or liver tissues (b & c) were run in the capillary western blotting system (Jess) and detected using a rabbit anti-CYP2D6 antibody followed by HRP-conjugated anti-rabbit secondary antibody. As an internal control (b & c), β-actin was also detected using a mouse anti-β-actin antibody followed by a NIR-conjugated anti-mouse secondary antibody. 2D6-F, full length CYP2D6; 2D6-S, CYP2D6ΔE3 isoform. Ref, pooled liver sample serves as a calibrator to normalize data from different runs. Two liver samples without CYP2D6 bands are homozygous for CYP2D6 null alleles (*4/*5 for L44 and *4/*4 for L35).
